# Methylome of human senescent hematopoietic progenitors

**DOI:** 10.1186/s40164-018-0123-8

**Published:** 2018-12-21

**Authors:** Stephen Capone, Anthony R. Colombo, Benjamin K. Johnson, Tim J. Triche, Giridharan Ramsingh

**Affiliations:** 1 Jane Anne Nohl Division of Hematology and Center for the Study of Blood Diseases, Keck School of Medicine of University of Southern California, Los Angeles, CA 90033 USA; 2 Center for Epigenetics, Van Andel Research Institute, Grand Rapids, MI USA

**Keywords:** Senescence, Hematopoietic stem and progenitor cells, Inflammation, Transposable elements, Endogenous retroviruses, Whole genome bisulfite sequencing, Methylation, CEBPβ, Transcriptome

## Abstract

Senescence, a state of permanent cell cycle arrest, can be induced by DNA damage. This process, which was initially described in fibroblasts, is now recognized to occur in stem cells. It has been well characterized in cell lines, but there is currently very limited data available on human senescence in vivo. We recently reported that the expression of transposable elements (TE), including endogenous retroviruses, was up-regulated along with inflammatory genes in human senescent hematopoietic stem and progenitor cells (HSPCs) in vivo. The mechanism of regulation of TE expression is not completely understood, but changes in DNA methylation and chromatin modifications are known to alter their expression. In order to elucidate the molecular mechanisms for TE up-regulation after senescence of HSPCs, we employed whole-genome bisulfite sequencing in paired senescent and active human HSPCs in vivo from healthy subjects. We found that the senescent HSPCs exhibited hypomethylated regions in the genome, which were enriched for TEs. This is the first report characterizing the methylome of senescent human HSPCs.

**Electronic supplementary material:**

The online version of this article (10.1186/s40164-018-0123-8) contains supplementary material, which is available to authorized users.

## Introduction

Aging is characterized by a progressive loss of organ function. The complex cellular process of stem cell aging likely contributes to the aging phenotype [1–3]. Cellular senescence, defined as a state of permanent cell cycle arrest, plays a distinct and important role in aging [4–6]. A plethora of stresses, such as telomere shortening, mitochondrial dysfunction, oxidative stress, DNA damage, and expression of oncogenes, can provoke senescence [7].

While the phenomenon of senescence was originally described in fibroblasts, it has now been shown to occur in stem and progenitor cells, with senescent hematopoietic, hepatic, endothelial, and skeletal muscle progenitor populations identified [8–15]. However, comprehensive molecular features of human senescent cells in vivo have not been well investigated. We recently identified and isolated circulating senescent HSPCs from healthy human subjects and showed that their transcriptome had elevated expression of transposable elements (TEs) [16].

Repetitive sequences comprise two-thirds of the human genome, out of which 50% are TEs [17]. TEs have been recognized to play an important role in the dynamics of species evolution by creating genetic diversity and their expression has also been shown to be up-regulated in both aging and senescence [18, 19]. We recently showed that the expression of TEs is suppressed in leukemic stem cells [20] which also appears to prime the cells for immune-mediated clearance via activation of the interferon pathway [21–23]. Hence, tight regulation of TEs appears crucial for homeostasis.

Recent studies have begun to explore the mechanisms regulating the expression of TEs. Epigenetic mechanisms, specifically DNA methylation and chromatin modifications, tightly regulate the expression of TEs [24–26]. DNA hypomethylation has been shown to activate TEs, which can subsequently lead to genomic instability, resulting in tumorigenesis [27, 28] or cellular clearance by the immune system [21, 22]. These studies were primarily performed using cell lines in vitro. We wanted to examine whether the TE induction observed in human senescent HSPCs in vivo was due to a similar loss of DNA methylation. For this, we performed whole-genome bisulfite sequencing (WGBS) on senescent and active human HSPCs in vivo and characterized their methylome.

## Materials and methods

### Human in vivo senescent CD34+ HSPC isolation

Human in vivo senescent CD34+ HSPC were obtained and isolated from healthy subjects as previously described [16]. Briefly, cells were drained from leukocyte reduction system cones collected from healthy platelet donors. HSPCs were enriched using the RosetteSep human progenitor enrichment cocktail (StemCell Technologies). Following a 20-min incubation with the enrichment cocktail at room temperature, mononuclear cells were isolated by density gradient centrifugation using Ficoll-Paque premium (GE Healthcare). The mononuclear cells were suspended in IMDM containing 100 nM bafilomycin A1 for 1 h at 37 °C, followed by incubation with C_12_FDG at 37 °C for 90 min. Then, the cells were washed with sorting buffer and stained with PE-conjugated anti-CD34, PE-Cy7-conjugated anti-CD38, and APC-conjugated anti-CD45 (eBiosciences) and subjected to fluorescence-activated cell sorting (FACS) (BD FACS Aria III). Dead cells were excluded based on propidium iodide staining. Samples were sorted for CD34+ and CD45dim+ cells to identify HSPCs and then gated for C_12_FDG staining for senescence-associated beta-galatosidase (SA-βgal) expression [29]. To identify CD38+ and CD38− populations, we used PE-Cy7-conjugated anti-CD38 antibody (eBiosciences). The protocol was approved by the Institutional Review Board (Protocol # IRB-HS-12-00693).

### WGBS and methylation analysis

Libraries for whole-genome bisulfite sequencing were generated from 5 ng of purified DNA from paired senescent and active CD34+ cells from 3 healthy human donors (33, 45, and 53 years of age) using the NuGen Ovation Ultralow MethylSeq Kit following the manufacturer’s protocol, for a total of 6 individual libraries. Samples were not pooled prior to library generation. Reads were aligned using Biscuit and Metilene was used for calling of differentially methylated regions. Motif analysis of DMRs was conducted using the PWMEnrich package with Hocomoco and Factorbook motif databases provided in the motifbreakR package [30].

### Chromatin conformation inference from WGBS data

Reads were aligned using biscuit and post-processed using biscuiteer [31] prior to compartment inference. Briefly, observed CpG loci were restricted to “open sea” regions and smoothed using a Dirichlet smoothing approach. Loci that had less than 3× coverage were set to values of NA and any locus that had greater than 50% NAs were removed from the dataset. Remaining NAs were imputed using k-nearest neighbors [32, 33]. Chromatin confirmation inference was performed as described previously [34] and implemented in compartmap [35].

## Results

### Senescent human HSPCs isolated from peripheral blood of healthy subjects

In order to assess if epigenetic alterations such as changes in DNA methylation contributed to increased expression of TEs in senescent HSPCs, we subjected paired senescent and active HSPCs from 3 healthy humans to WGBS. We employed a modified FACS protocol [16] to identify and isolate senescent and non-senescent HSPCs (CD34+ CD45dim+ cells) from peripheral blood of healthy platelet donors. Senescence-associated β-galactosidase (SA-β-gal) was used as a senescence marker [12–15, 36, 37] to identify and isolate circulating senescent HSPCs as previously described [16]. Using this technique, we isolated between 2800 and 4800 senescent HSPCs and 250,000 to 360,000 non-senescent HSPCs from each of our three individual donors. All samples were standardized to an input of 5 ng prior to WGBS library generation.

### Senescent human HSPCs display focal loss of DNA methylation

**Fig. 1:**
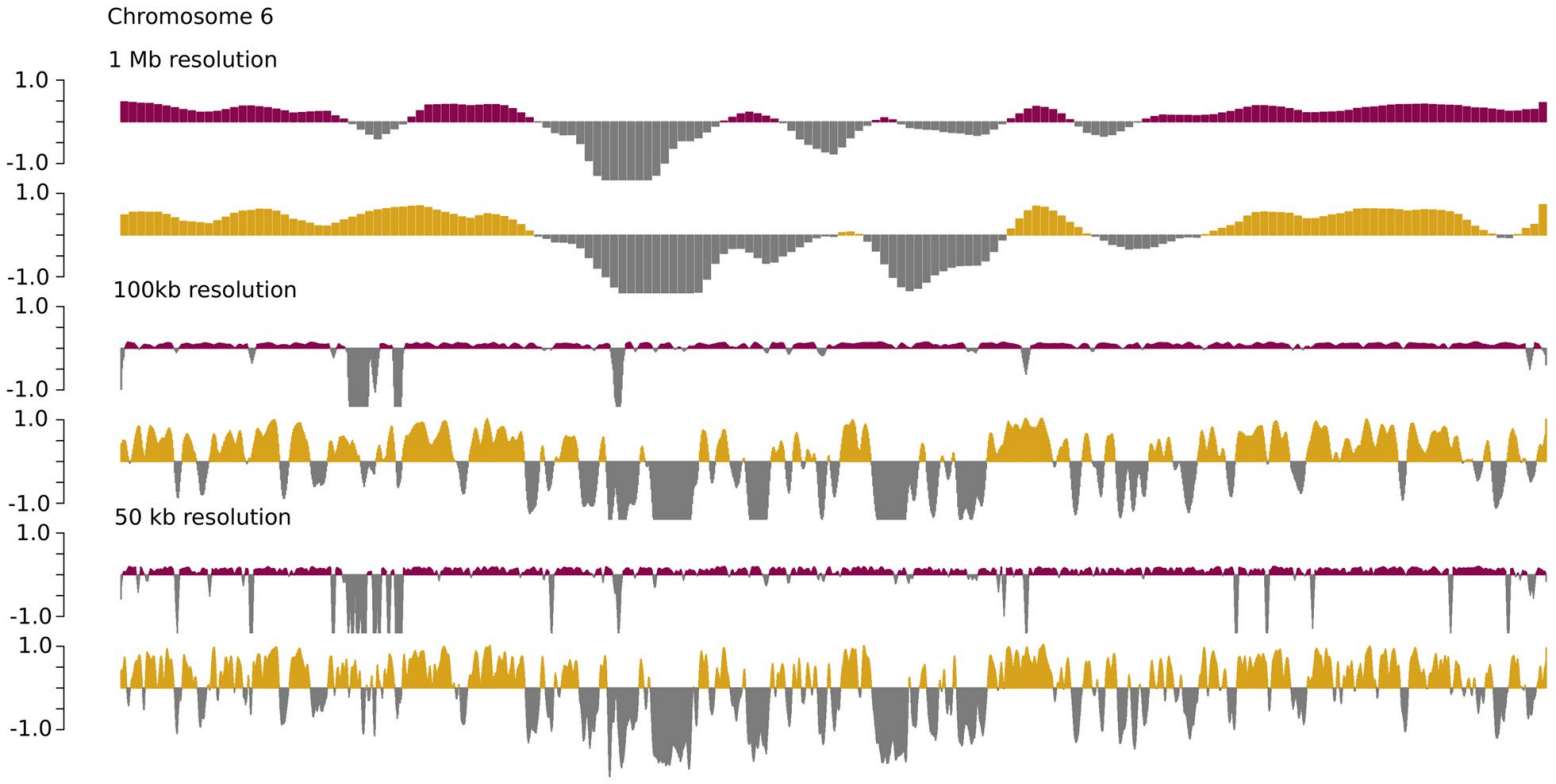
Multiresolution chromatin confirmation. Multi-resolution chromatin conformation inference for non-senescent (red/gray) and senescent (yellow/gray) HSPCs across chromosome 6 reveals representative genome-wide changes in open/closed compartments between cell types. The 50 kb resolution shows widespread focal chromatin accessibility across chromosome 6, encompassing regions rich in TEs. Open chromatin (negative eigenvalues); closed chromatin (positive eigenvalues)

**Fig. 2:**
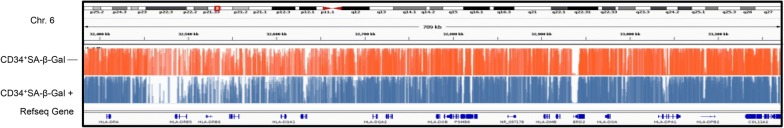
Whole genome bisulfite sequencing of senescent HSPCs. Focal loss of DNA methylation observed in senescent HSPCs obtained through whole genome bisulfite sequencing. Senescent population (CD34+ SA-β-Gal –) is represented in orange, with non-senescent population (CD34+ SA-β-Gal +) shown in blue. This shows alterations in methylation along a representative segment of chromosome 6, with focal hypomethylation of gene promoter regions of HLA-DRB5 and HLA-DRB6

**Fig. 3:**
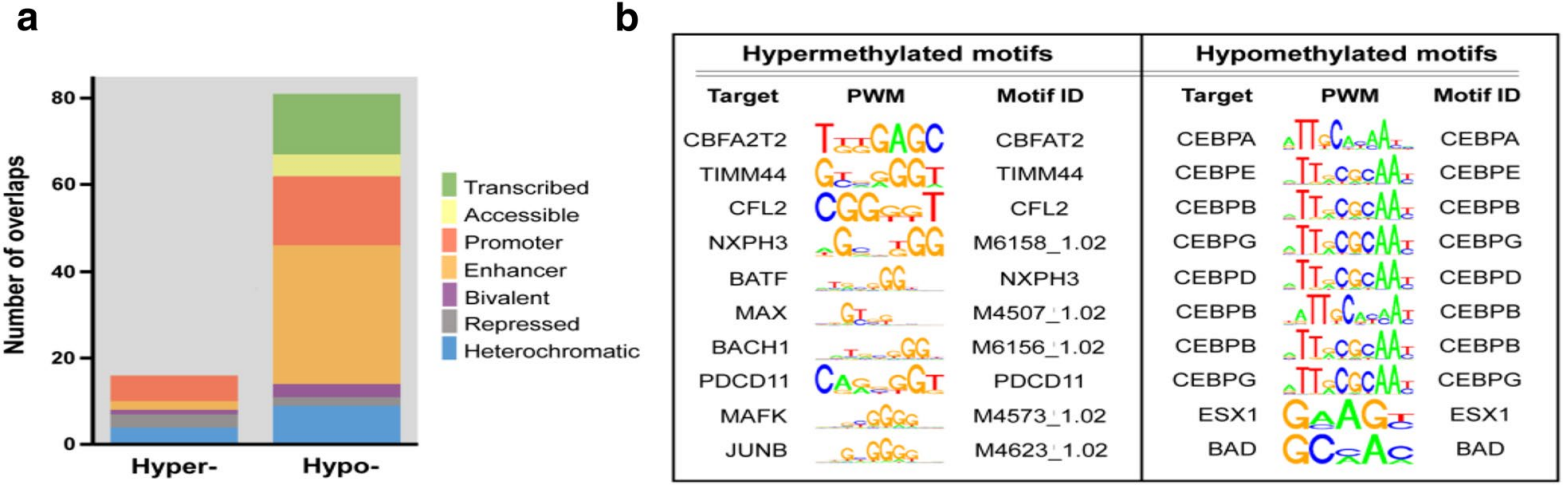
Characterization of the differentially methylated regions in senescent HSPCs. **a** Mapping of hypo- and hyperDMRs to chromatin states of CD34+ cells. Hypomethlation is spread diversely throughout different regions of the genome, with promoter and enhancer regions showing a propensity for hypomethylation. **b** Motif enrichment analysis of statistically significantly DMRs shows high concordance of both hypermethylated and hypomethylated regions of the genome, with selected targets noted. Hypomethylated regions show a clustering of CEBP enhancer regions

WGBS yielded 7.5× genome coverage. Using the 2-dimensional Komogorov-Smirnov approach implemented in Metilene with cutoffs of 10% minimum difference, 10 CpGs and 10% FDR, we identified 61 differentially methylated regions (DMRs) in senescent vs. active HSPCs, of which 51 were hypo-methylated (hypoDMRs) and 10 hyper-methylated (hyperDMRs) (Additional file 1: Table S1). Multi-resolution chromatin conformation inference for non-senescent and senescent HSPCs revealed representative genome-wide changes in open/closed compartments between cell types (Fig. 1). DNA methylation changes in the senescent cells were focal rather than global (Figs. 1 and 2). By mapping the hypoDMRs to chromatin states using ChIP-seq data of primary human CD34+ cells, we found the majority of DMRs to overlap with transcriptional enhancers (Fig. 3b). CCAAT/enhancer binding proteins (CEBPA/B/G) were the dominant motif (8/10 top hits) in hypoDMRs (Fig. 3b). CBFA2T2, TIMM44, and Myc-associated factor X were among the top hits in hyper DMRs (Fig. 3b).

### Enrichment of repetitive elements in the hypoDMRs of senescent HSPCS

**Fig. 4:**
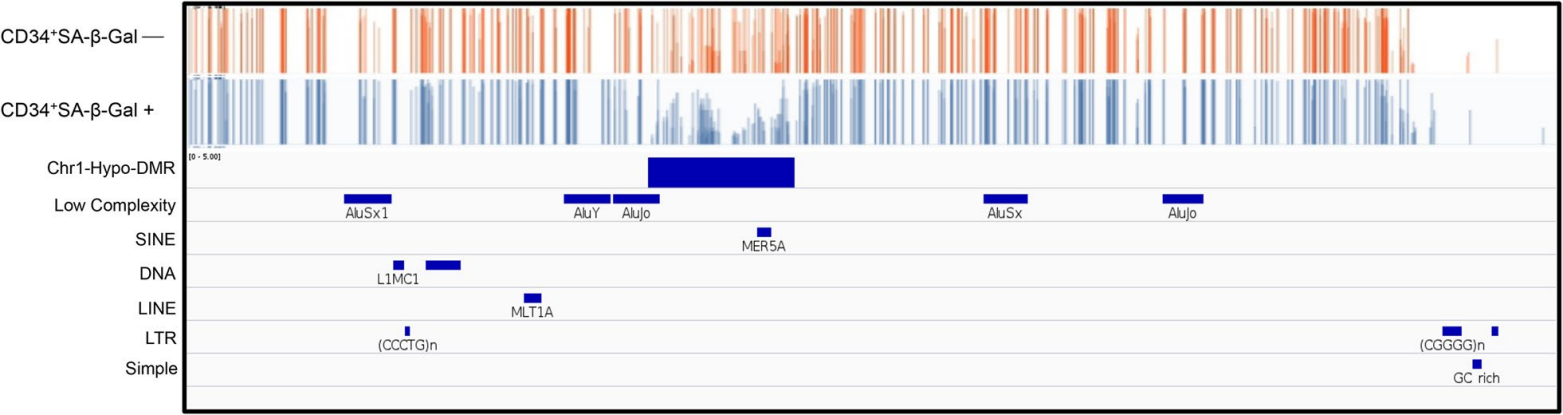
Regions with loss of methylation overlapped with the repeat elements in the genome. Senescent population (CD34+ SA-β-Gal –) is represented in orange, with non-senescent population (CD34+ SA-β-Gal +) shown in blue in this representative region of chromosome 6 (158507720:158508419) and region of hypomethylation overlapping with transposable element

Next we examined whether the hypoDMRs correlated with the repeat regions in the genome. Interestingly, all of the hypoDMRs (51/51) but only 4/10 of the hyperDMRs overlapped with repeat elements (Fig. 4, Additional file 2: Figure S1 and Additional file 3: Table S2). Based on the observation that ~ 50% of the genome is repetitive and that an overlap may equally likely affect hyper- and hypo-DMRs, Fisher’s Exact test showed a significantly increased occurrence of repeat elements in the hypoDMRs (P < 10^−6^). This led us to conclude that senescent HSPCs display focal loss of DNA methylation in the repetitive DNA-containing DMRs.

## Discussion

This is the first study to perform WGBS in human senescent cells in vivo. Previous WGBS studies on replicative senescence in cell lines showed global genome-wide loss of methylation [38]. Cruickshanks et al. [38] suggest that methylation signatures in senescence are fixed once the cell cycle ceases, and are therefore a reflection of previous epigenetic events. Our study using in vivo senescent HSPCs showed only focal loss of methylation, suggesting the mode of senescence in in vitro replicative senescent cells and in human in vivo senescent HSPCs were likely different. DNA methylation changes that accumulate over time during cell passage in vitro may be a reason for this difference. We speculate that the senescence in HSPCs was likely due to a stress response, such as oxidative stress. Our study showed that the hypoDMR regions were enriched for both TEs and enhancer marks. This finding is consistent with prior studies showing hypomethylated TE-rich genomic regions containing enhancer marks [25, 26]. TEs are known to play a significant role in regulation of gene expression [39]. The physical proximity of the TE and enhancers regions is possibly a result of co-evolution. Future studies on how TEs cooperate with the nearby enhancer regions to modulate gene expression are warranted.

Several chromatin modifiers including CTCF, BORIS, DDM1, LSH1, KDM1A and transcription factors like p53, SIRT1, FOXA1, SP1 have been shown to maintain the TEs in a dormant state [40–42]. We found that CEBP binding sequences were enriched in the focally hypomethylated regions of the genome. Previous studies have shown methylation-specific increases in DNA binding affinity for CEBPβ [43–47], which also plays a significant role in regulating senescence associated secretory phenotype (SASP), an inflammatory phenotype known to occur with senescence induction [43–47]. It is possible that CEBPβ regulates TE expression in a methylation-specific manner, a mechanism that needs to be explored.

The major limitation of this study is the low sequencing depth, which may lead to the relatively low number of DMRs identified. We hypothesize that future deeper sequencing with high coverage will help elucidate additional DMRs, helping unlock additional upregulation of TEs. Because of the low depth of sequencing, it is possible that not all DMRs met the stringent requirements for our study, yet are still contributing to the overall upregulation of TEs. It is also important to note that hypomethylation may not be the only factor contributing to the upregulation of TEs. Histone modifications are a major mechanism that may also play a role in this overexpression of TEs and should be further investigated. These changes can be evidenced by the widespread focal opening of chromatin not solely accounted for by DMRs. High resolution mapping of chromosome 6 shows increasing numbers of focal open regions in senescent HSPCs when compared to non-senescent. This is consistent with a widespread focal opening of chromatin, which could lead to increased TE expression.

Recent studies have elucidated the role of TEs in various pathologies, such as motor neuron disease, autoimmune diseases and cancers [48–55], motivating a deeper understanding of the dysregulation mechanisms of TEs. Understanding the regulation of TE expression could enable better understanding of the pathophysiology of the disease, facilitating the development of better treatment options.

Dysregulation of TEs has also been implicated in accelerated aging in mouse models of senescence. Loss of methylation in TE-rich regions of the genome has been shown in both mice and human aging [56–58]. Recent studies have shown that hypomethylating agents in cell lines induce the expression of TEs, which causes activation of the viral recognition pathway and inflammatory gene expression [21, 22]. We speculate that similar mechanisms may underlie the inflammatory phenotype seen in senescence, warranting further mechanistic studies in senescence exploring the link between hypomethylation, activation of TE expression and immune activation.

## Additional files

**Additional file 1.** Compiled list of differentially methylated regions in senescent vs. active HSPCs, including 51 hypomethylated and 10 hypermethylated regions.

**Additional file 2.** Chromosomal locations of hypoDMRs overlapping with repeat elements. The type of repeat and family of repeats are noted.

**Additional file 3.** A representative region of chromosome 6 showing the characteristic overlap of hypoDMRs with repeat elements.
