# Supplementary material for: Methylome of human senescent hematopoietic progenitors
Source: Exp Hematol Oncol. 2018 Dec 21;7:32. doi: 10.1186/s40164-018-0123-8 (PMC6302458; doi:10.1186/s40164-018-0123-8)
Supplement: Supplementary file 2 — Additional file 2. Chromosomal locations of hypoDMRs overlapping with repeat elements. The type of repeat and family of repeats are noted. [file 40164_2018_123_MOESM2_ESM.pdf]

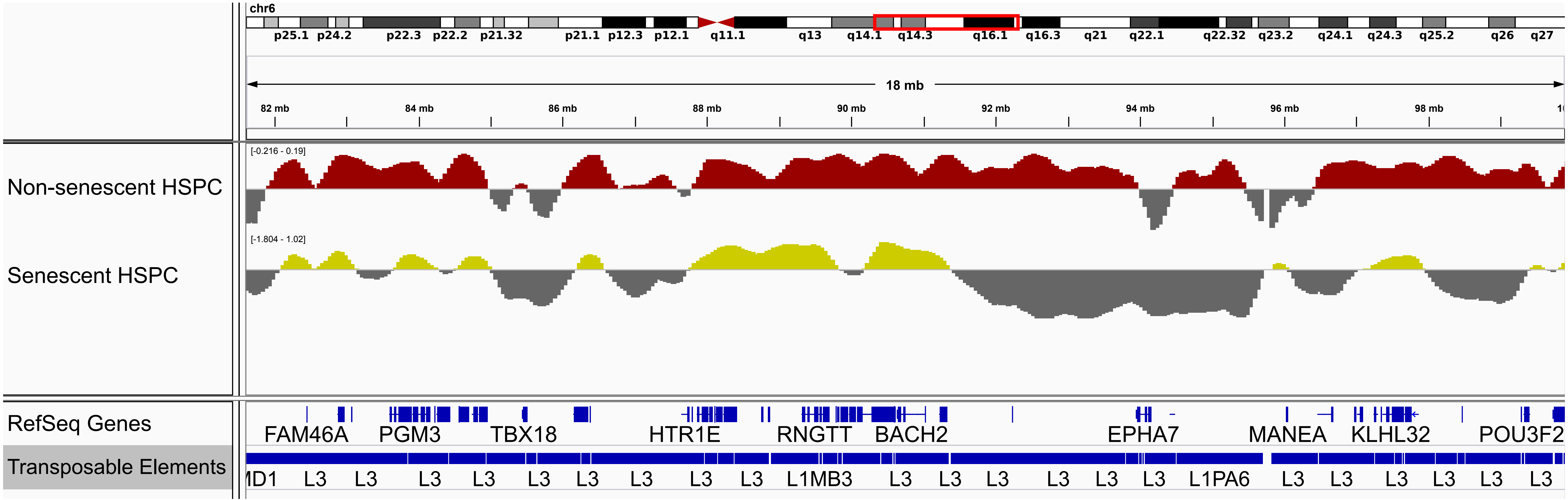
chr6
p25.1 p24.2 p22.3 p22.2 p21.32 p21.1 p12.3 p12.1 q11.1 q13 q14.1 q14.3 q16.1 q16.3 q21 q22.1 q22.32 q23.2 q24.1 q24.3 q25.2 q26 q27
18 mb
82 mb 84 mb 86 mb 88 mb 90 mb 92 mb 94 mb 96 mb 98 mb
Non-senescent HSPC
[-0.216 - 0.19]
Senescent HSPC
[-1.804 - 1.02]
RefSeq Genes
FAM46A PGM3 TBX18 HTR1E RNGTT BACH2 EPHA7 MANEA KLHL32 POU3F2
Transposable Elements
L3 L3 L3 L3 L3 L3 L3 L3 L3 L1MB3 L3 L3 L3 L3 L3 L3 L1PA6 L3 L3 L3 L3 L3 L3
